# Who Sets the Aggressive Popularity Norm in Classrooms? It’s the Number and Strength of Aggressive, Prosocial, and Bi-Strategic Adolescents

**DOI:** 10.1007/s10802-019-00571-0

**Published:** 2019-07-20

**Authors:** Lydia Laninga-Wijnen, Zeena Harakeh, Jan Kornelis Dijkstra, René Veenstra, Wilma Vollebergh

**Affiliations:** 1 Department of Interdisciplinary Social Sciences, Utrecht University, Padualaan 14, 3584 CS Utrecht, The Netherlands; 2 TNO, Child Health Department, Leiden, The Netherlands; 3 Department of Sociology, University of Groningen, Groningen, The Netherlands

**Keywords:** Aggression, Prosocial behavior, Popularity norm, Social dominance, Bi-strategic, Popularity hierarchy

## Abstract

Previous work has shown that during adolescence, classrooms vary greatly in the extent to which aggression is rewarded with popularity (the ‘popularity norm’). Aggressive popularity norms may promote the proliferation of aggression and negatively affect the classroom climate. It is, however, unknown how these norms emerge in the first place. This longitudinal study therefore investigated whether aggressive popularity norms can be predicted by the classroom composition of students. We examined whether the prevalence of six student types - socially and non-socially dominant prosocial, aggressive, and bi-strategic adolescents (adolescents who are both highly prosocial and aggressive) - contributed to the norm by establishing a popularity hierarchy: strong classroom asymmetries in popularity. We collected peer-nominated data at three secondary schools in the Netherlands (SNARE-study; *N*_students_ = 2843; *N*_classrooms_ = 120; 51.4% girls; *M*_age_ = 13.2). Classroom-level regression analyses suggest that the classroom percentage of socially dominant aggressive and bi-strategic students predicted higher aggressive popularity norms, both directly and by enhancing the classrooms’ popularity hierarchy. Instead, the presence of non-socially dominant aggressive students and socially dominant prosocial students contributed to *lower* aggressive popularity norms. Socially dominant prosocial students also buffered against the role of socially dominant aggressive adolescents in the aggressive popularity norm (moderation), but not against bi-strategic adolescents’ role. Our findings indicate that interventions aimed at reducing aggressive popularity norms should first and foremost take the composition of classrooms at the start of the school year into account; and should not only encourage prosocial behavior, but also actively discourage aggression.

Ushered in with pubertal and social changes, adolescents increasingly attach value to being popular among their peers (Steinberg, 2007; Koski, Xie, & Olson, 2015; LaFontana & Cillessen, 2010). Behaviors that are rewarded with popularity can be seen as salient and valuable tools to gain or maintain a high position in the peer group (Hartup, 1996), and may therefore form an important norm for adolescents: a guideline prescribing how they should behave in order to fit in with expectations of the peer group and to prevent being a social misfit (Wright, Giammarino, & Parad, 1986). Previous work has shown that classrooms vary in the extent to which aggression is associated with popularity (e.g. the popularity norm). Aggressive popularity norms have been shown to emerge rapidly in classrooms and to be quite persistent, remaining relatively stable across the school year (Laninga-Wijnen et al. 2018). Aggressive norms enhance conditions for the proliferation of aggression (Laninga-Wijnen, Harakeh, Steglich, Dijkstra, Veenstra, & Vollebergh, 2018), which may have an adverse impact on the classroom environment. Indeed, classrooms with aggressive popularity norms were found to be characterized by higher levels of peer rejection and victimization as well as lower academic performance and less positive feelings about school among students (Dijkstra & Gest, 2015). Whereas previous research has focused mainly on the consequences of popularity norms, little is known about the origins of these norms. In order to prevent these norms from emerging, schools may benefit from a better understanding of which factors are associated with the development of these norms at the start of the school year. As popularity norms are established so quickly, it may be that they are related to the presence of particular types of students in a certain class from the moment the class is formed. If so, classroom composition may be an important factor in the formation of classroom norms.

Different types of students can be identified based on the extent to which they endorse aggressive or prosocial behavior, or a combination of both (bi-strategics; see Resource Control Theory, Hawley 1999; McDonald et al. 2015; 2015). Based on Social Impact Theory (SIT, Latané 1981), the formation of norms may be a function of 1) the *number of people* endorsing certain behaviors; 2) the *social dominance* (strength) of those enacting these behaviors, referring to power-related characteristics such as leadership qualities or resource control; and 3) the *immediacy* of these people, i.e. the closeness in space or time (Latané and Wolf 1981). Adolescents spend much of their time at school in the immediate proximity of their classmates (closeness of people); we will therefore focus on norm formation in the classroom context, by examining whether aggressive popularity norms are predicted by the number (percentage) and strength (social dominance) of prosocial, aggressive, and bi-strategic students in a particular class. Additionally, we will examine in what ways the presence of these student types may contribute to the popularity norm (mediation). We will investigate whether certain types of students would strengthen the formation of a strong popularity hierarchy (asymmetries in popularity within a classroom), which – following a balance of power perspective (Garandeau et al. 2014; Laninga-Wijnen et al. 2019) – may enhance the valence of aggression in classrooms (higher aggressive popularity norms; Ahn et al. 2010).

## The Number and Strength of Aggressive, Prosocial and bi-Strategic Adolescents

Several social psychological theories – including SIT – suggest that *numbers* and influence on norms go hand in hand (for a review, see Bond 2005). These theories almost exclusively focus on the influence of numerical *majorities*: the more people who endorse a certain behavior, the more influence they will exert over which behaviors are considered appropriate and normative (Latané 1981). Numerical majorities have more resources to reward conformers and punish deviants (resulting in compliance), and a greater capacity to provide information about reality (resulting in conformity; Bond 2005; Deutsch & Gerrard, 1955).

However, according to Moscovici and Faucheux (1972), even a numerical *minority* of people can exert influence over what behaviors are considered valuable and normative, for instance when individuals constituting this minority are *consistent in behavior* (e.g. show high levels of a certain behavior, or show this behavior towards multiple peers), as this demonstrates that this small group of individuals is confident and committed to enacting this behavior (Moscovici and Faucheux 1972; Moscovici and Nemeth 1974; Wood et al. 1994). In reply to Moskovici’s work (1971), Latané and Wolf (1981) stressed that SIT can also be applied to numerical minorities; hence the extent to which behavior is seen as salient and normative may, in this case, depend on the *number* of people constituting a minority.

Latané and Wolf (1981) additionally emphasized the importance of a numerical minority’s *strength*: people may only contribute to the norm when they combine their behavior with social dominance, which refers – among other things – to centrality and leadership features (Latané 1981; Hawley 1999). First, social dominance can be seen as an evolutionary adaptive characteristic (Berry 2000; Hawley 1999) which invokes respect and admiration; as a consequence, behaviors of socially dominant adolescents are seen in a positive light and may become a norm (Dijkstra, Lindenberg, Verhulst, Ormel, & Veenstra, 2009). Second, socially dominant adolescents may portray themselves as leaders and role models (Ellis and Zarbatany 2007; Waasdorp et al. 2013). Their behaviors acquire valence as conforming to these behaviors may prevent rejection by peers and enhance the chance of leaders’ approval (Farmer et al. 2003). Importantly, SIT is mainly about *same-*behavior processes, suggesting that the formation of norms related to a certain behavior (e.g. aggression) depends on the number and social dominance of individuals displaying that particular kind of behavior (aggression). Yet not *all* adolescents who are aggressive may score highly on social dominance. For instance, earlier research on seventh and eighth grade students identified two types of aggressive youth: a first group contained non-socially prominent aggressors relegated to peripheral positions in the peer group; a second group contained highly central, aggressive leaders (Troubled versus Toughs; Farmer et al. 2003). Based on SIT (Latané 1981) and Moscovici’s theory on numerical minorities (1972), it might therefore be expected that classrooms with relatively more socially dominant, consistently aggressive individuals would be characterized by higher aggressive popularity norms. If consistently aggressive individuals (Moskovici, 1972) lack social dominance (Latané and Wolf 1981), they may lack the power to contribute to the norm.

Although SIT (Latané 1981) is mainly concerned with same-behavior processes, it can be reasoned that *cross-*behavior processes may occur as well. More specifically, individuals displaying *related* behaviors such as prosocial behavior (Obsuth et al. 2015) may also play a role in aggressive popularity norms (Laninga-Wijnen et al. 2019); and the extent to which they have the power to do this may also depend on their social dominance (Latané and Wolf 1981). Prosocial behavior by students may either decrease or increase the valence of aggression, depending on the type of student (e.g. bi-strategic *or* prosocial individuals) using it. For example, the prosocial behavior of socially dominant *bi-strategic* individuals may increase rather than decrease the valence of aggression, as these well-adapted ‘Machiavellians’ are assumed to deliberately use their prosocial behavior to mitigate the negative consequences of their aggression and to hide it from teachers (Hawley 2003), making aggression more attractive. As a result, a higher number of socially dominant bi-strategic individuals in classrooms may be associated with higher aggressive popularity norms. In contrast, socially dominant, solely prosocial adolescents (‘models’; Berger et al. 2015) may contribute to a safe, friendly, harmonious classroom environment (Jennings and Greenberg 2009) where aggression is perceived as non-adaptive (Chang 2004), resulting in lower aggressive popularity norms. In addition, socially dominant prosocial individuals may provide a *buffer against* the role of socially dominant aggressive or bi-strategic individuals in aggressive popularity norms (moderation effect), as they may provide a counterweight to aggression (Obsuth et al. 2015) and model a valuable alternative – being (solely) prosocial – to gain access to valuable social or material resources (Berger et al. 2015; Ellis et al. 2016; Hawley 1999;). By contrast, *non-socially dominant* prosocial and bi-strategic students may not be important for the popularity norm, even when their number is high; as they lack the strength to contribute to the classroom environment (Latané and Wolf 1981).

## Popularity Hierarchy as Underlying Mechanism for how Different Types of Students Contribute to Aggressive Popularity Norms

One way in which socially dominant aggressive and bi-strategic individuals may contribute to higher aggressive popularity norms is by enhancing the classrooms’ popularity hierarchy (e.g. classroom asymmetries in popularity). Socially dominant aggressive and bi-strategic students are thought to use their aggression in a strategic, manipulative way (Farmer et al. 2003; Hawley 2003), allowing them to gain a higher status in the peer group *at the expense of* the status of others. This may enhance status discrepancies in the classroom. A higher number of these socially dominant aggressive and bi-strategic individuals may therefore be related to higher within-classroom variation in individuals’ status such as popularity, also referred to as a ‘strong popularity hierarchy’ (typically measured as the standard deviation of students’ popularity within classrooms; Zwaan et al., 2013; Garandeau et al. 2011). According to a *balance of power perspective* (Garandeau et al. 2014; Juvonen et al. 2006), strong asymmetries in popularity induce a *power imbalance,* which facilitates abuse of power through aggression on the part of popular peers. Moreover, when the benefits associated with popular status are not equally available (Hawley 2003), adolescents may compete for popularity more strongly, and aggression may be seen as a valuable means of gaining or maintaining popularity (Garandeau et al. 2011). In line with this reasoning, one previous study (Laninga-Wijnen et al. 2019) demonstrated that a strong popularity hierarchy predicted higher aggressive popularity norms over time. Nevertheless, to date it is unknown what types of students contribute to the popularity hierarchy, and whether popularity hierarchy can be seen as an explanatory mechanism (e.g. mediator) for the association between classroom percentages of student types and the popularity norm. We would expect classrooms with more socially dominant aggressive and bi-strategic individuals to be characterized by stronger popularity hierarchies, and hence, higher aggressive popularity norms. Instead, classrooms with more socially dominant prosocial individuals may represent relatively democratic environments with a shared balance of power, as these prosocial leaders may set a norm for showing behaviors *benefitting others* rather than lowering others’ status (Eisenberg et al., 2006). A higher number of socially dominant prosocial individuals may therefore be associated with a less strong popularity hierarchy (e.g. more egalitarian classrooms), which in turn may relate to lower aggressive popularity norms. We did not have clear expectations regarding how the number of non-socially dominant individuals may contribute to the popularity hierarchy, and we therefore explored their potential role.

## Classroom Demographic Characteristics and Aggressive Popularity Norms

In addition to the role of the number and strength of aggressive, prosocial and bi-strategic students, general demographic classroom characteristics – sex proportion, classroom size, school year and education level – could also contribute to the formation of aggressive popularity norms. As aggression is more prevalent among boys than among girls, and is generally described as a reputationally salient characteristic of boys (Hartup, 1996), a higher proportion of boys in the classroom may predict higher aggressive popularity norms. Classroom size predicted lower aggressive popularity norms in one study (Garandeau et al. 2011), but was unrelated to aggressive popularity norms in another study (Gest & Rodkin, 2011); hence we explored the role of classroom size in popularity norms. As aggression becomes a more important associate of popularity during early adolescence (Cillessen & Mayeux, 2004), partly due to the ‘maturity gap’ (Moffitt, 1993), we expected that the aggressive popularity norm would increase with higher grades. We also expected that aggressive popularity norms would be less likely to emerge at higher education levels, as attitudes towards achievement are more likely to be positive and therefore hardly compatible with aggression, which should decrease the valence of aggression in these contexts (Garandeau et al. 2011).

## The Present Study

This study aims to examine whether and how the *number* of six types of students – that is, socially and non-socially dominant aggressive, prosocial, and bi-strategic students – contributes to the aggressive popularity norm. Based on SIT (Latané and Wolf 1981), we expect the role of these student types to be dependent on the number and strength of these students. Only socially dominant students – but *not* non-socially dominant students – may have the power to set the norm: a higher number of socially dominant aggressive and bi-strategic adolescents may enhance aggressive popularity norms, whereas a higher number of socially dominant prosocial adolescents may be associated with lower aggressive popularity norms. Moreover, we expect socially dominant prosocial adolescents to provide a buffer against the role of socially dominant aggressive or bi-strategic individuals in predicting the aggressive popularity norms (moderation effects). We will predict popularity norms at the start of the school year (T1), and at the end of the school year (T3; after controlling for norms at T1). As popularity norms have been found to emerge quickly (Laninga-Wijnen et al. 2018), we also examine whether the number of different student types contributes *indirectly* to popularity norms at T3, via their role in popularity norms at T1. In addition, we examine whether popularity hierarchy can be a mediating factor: We expect the number of socially dominant aggressive and bi-strategic individuals to enhance a classroom’s popularity hierarchy and, in turn, to contribute to higher aggressive popularity norms, whereas the number of socially dominant prosocial individuals may be related to more egalitarian classrooms and consequently result in lower aggressive popularity norms.

## Method

### Participants and Procedure

We approached all first and second-year students at two secondary schools in the Netherlands (comparable to Grade 7–8 in the U.S.) to participate in the SNARE project at the start of the 2011–2012 school year (Cohort 1). For the next school year, 2012–2013, a second cohort of first-year students entering these secondary schools was asked to participate in the project (Cohort 2). A third cohort of first, second and third-year students was approached at another school in the 2016–2017 school year. Data were collected at three points during one school year: in the autumn, winter, and spring. In this study, data were used from the first and third measurement wave (T1 and T3). Before data collection started, students and their parents received an information letter explaining the goal of the study and offering the possibility to decline participation. Parents who did not wish their children to participate in the study were asked to indicate this, and students were told that they could opt out of their participation at any time. The survey was completed in the classroom under the supervision of a research assistant, using the Bright Answer socio-software (SNARE software, 2011). The study was approved by the Ethical Internal Review Board of one of the participating universities (Utrecht University), and the privacy and confidentiality of students’ data were guaranteed.

Of the 2914 students approached, 71 (2.4%) declined to participate for several reasons (e.g. the student was dyslectic, or parents considered the questionnaire too time-consuming). The final sample comprised 2843 participants from 120 classrooms, with 12–30 participants per classroom (*M* = 23.69). Around 54% of the participants were first-year students (grade 7), 37% were second-year students (grade 8) and 9% were third-year students (grade 9). Participants’ ages ranged from 11 to 17 years (*M* = 13.17, *SD* = 0.80); 51.4% were girls. About 40% were in lower secondary education (i.e. preparatory secondary school for technical and vocational training); 60% were in higher secondary education (including senior secondary vocational and pre-university education). The majority of the sample (approximately 85%) were native Dutch.

### Measures

All measures were based on peer nominations, assessed by asking participants questions about their classmates. Participants could nominate an unlimited number of same-sex and cross-sex peers. They also had the option of not selecting anyone for an item. For all items, the total number of nominations received was divided by the number of nominators, so that scores represented the proportion of classmates who had nominated an individual adolescent.

#### Aggressive Behavior

Peer-perceived aggressive behavior was assessed using four within-classroom peer nominations concerning aggressive behavior: “Who quarrels and/or initiates fights with you?”; “Who sometimes spreads rumors or gossip about you?”; “Who makes fun of others?”; and “Who bullies you?”. Principal component factor analyses showed that these four items loaded on one factor, explaining 61.6% of the variance at T1 (factor loadings varying from .74 to .83) and 64.1% of the variance at T3 (factor loadings ranging from .78 to .83). These four items were therefore averaged to create a scale for aggressive behavior at both T1 and T3. This scale represented the average proportion of peers who nominated a particular adolescent as aggressive using the four items, which could vary from 0 (nominated by nobody on the four items) to 1 (nominated by everyone on all four items). Cronbach’s alphas were *α*_*T1*_ = .72, and *α*_*T3*_ = .73, indicating adequate internal consistency.

#### Prosocial Behavior

Peer-perceived prosocial behavior was assessed using three items (see also Laninga-Wijnen et al. 2018): “Who gives others the feeling that they belong to the group?”; “Who helps others by giving good advice?”; and “Who help you with problems (e.g. with homework, repairing a flat tire, or when you feel down)?” Principal component factor analysis showed that these three items represented one factor, explaining 64.1% of the variance (factor loadings ranging from .75 to .88) at T1 and 74.7% of the variance at T3 (factor loadings ranging from .84 to .86). The average of these three items was used as a scale for peer-perceived prosocial behavior, both at T1 and T3. This scale represented the average proportion of peers who nominated a particular adolescent as prosocial using the three items, which could vary from 0 (nominated by nobody on the three items) to 1 (nominated by everyone on all three items). Cronbach’s alphas of the resultant scale were *α*_*T1*_ = .72, and *α*_*T3*_ = .83, indicating the scale to be internally consistent.

#### Social Dominance

In order to measure social dominance, three peer-nominated items were used: “Who makes others follow their plans?” “Who gets attention from others?” and “Who do others choose to lead the group?” Principal component factor analysis indicated that these three items represented one factor explaining 72.6% of the variance (factor loadings ranging from .84 to .87). We calculated the average of these three items at T1 as a scale for peer-perceived social dominance. This scale represented the average proportion of peers who nominated a particular adolescent as socially dominant using the three items, which could vary from 0 (nominated by nobody on the three items) to 1 (nominated by everyone on all three items). Cronbach’s alpha of this scale was *α*_*T1*_ = .75.

#### Construction of Student Types

Our analyses were carried out at classroom level, as our aim was to predict classroom-level outcomes. As we were interested in combinations of social dominance and behaviors *within persons*, our first step was to group our participants based on these combinations *within persons*. In order to identify the six types of students –socially dominant aggressive, prosocial and bi-strategic individuals, and *non-*socially dominant aggressive, prosocial and bi-strategic individuals – we needed a cut-off to define high aggression, prosocial behavior or social dominance. As Resource Control Theory inspired our study, we initially aimed at replicating the cut-off presented by Hawley (1999), who divided her sample into thirds in order to distinguish between aggressive, prosocial and bi-strategic controllers (see McDonald et al., for a similar approach). However, when we applied this criterion to our data, adolescents who were nominated only once on one aggression item would still be regarded as highly aggressive. As the work of Moscovici (1974) emphasizes the importance of a certain degree of consistency in behavior, we aimed at categorizing adolescents as aggressive, prosocial or socially dominant when they were chosen at least twice on items belonging to these scales (i.e. nominated by at least two classmates, or on at least two items). In order to meet this ‘consistency’ criterion, we decided to use the 75th percentile as a cut-off for defining high levels of aggression, prosocial behavior and social dominance; hence introducing a somewhat stricter criterion than Hawley (1999).

**Fig. 1:**
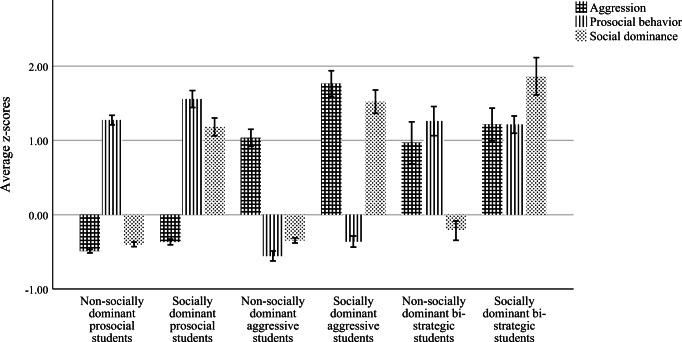
Behavioral profiles of six student types based on the 75th percentile of *z*-standardized aggression, prosocial behavior, and social dominance scores

For aggression, prosocial behavior and social dominance scores, students were assigned a ‘1’ if they scored above the 75th percentile and a ‘0’ if they scored below this 75th percentile. Based on these three binary variables, six types of students at T1 were distinguished across the whole sample. For instance, socially dominant bi-strategic students scored a ‘1’ on all binary variables (i.e. scored above the 75th percentile for prosocial behavior, aggression *and* social dominance). By contrast, non-socially dominant bi-strategic students scored above the 75th percentile for aggression and prosocial behavior, but not above the 75th percentile for social dominance. Figure 1 presents the behavioral and social dominance characteristics of these six types of students, and Table 1 provides information on how they varied by composition.

**Table 1:** ANOVA test of differences in aggression, prosocial behavior, and social dominance at the baseline (T1) between the six student types

	Socially dominant aggressive students (*N* = 251)	Socially dominant bi-strategic students (*N* = 104)	Socially dominant prosocial students (*N* = 195)	Non-socially dominant aggressive students (*N* = 315)	Non-socially dominant bi-strategic students (*N* = 36)	Non-socially dominant prosocial students (*N* = 388)
	*M (SE)*	*M (SE)*	*M (SE)*	*M (SE)*	*M (SE)*	*M (SE)*
Aggression	.10 (.06)^c^	.08 (.05)^b^	.01 (.01)^a^	.07 (.04)^b^	.07 (.03)^b^	.01 (.01)^a^
Prosocial behavior	.09 (.04)^c^	.19 (.04)^b^	.22 (.05)^a^	.08 (.04)^d^	.20 (.04)^ab^	.20 (.04)^b^
Social dominance	.16 (.08)^c^	.18 (.09)^d^	.13 (.06)^b^	.03 (.02)^a^	.04 (.03)^a^	.03 (.02)^a^
Boy	68.5%	46.2%	34.4%	67.0%	33.3%	18.0%
% Western	79.3%	88.5%	86.7%	78.7%	88.9%	86.9%
Age (years)	13.35 (.89)^b^	13.17 (.70)^abc^	13.07 (.76)^a^	13.08 (.71)^c^	13.15 (.74)^abc^	13.28 (.81)^ab^

For each row, parameters with different superscripts differ significantly from each other (post-hoc test with Bonferroni correction)

### Class-Level Variables

#### Demographic Variables

We included four demographic variables to control for their potential effect on the aggressive popularity norm, in line with previous work (Laninga-Wijnen et al. 2019): class size, educational level, grade, and sex proportion. Class size was measured as the total number of participating adolescents in a classroom. Education level was included as a binary variable, with ‘0’ referring to lower education levels (including preparatory secondary school for technical and vocational training) and ‘1’ referring to higher education levels (senior secondary vocational and pre-university education). For a more detailed description of the tracked education system in the Netherlands, see Gremmen et al. (2017). Secondary school year (grade) varied from first to third year. Sex proportion was calculated as the percentage of boys within a class, by dividing the number of participating boys by the total number of participants.

#### Aggressive Popularity Norms

Peer-nominated popularity was assessed by asking participants “Who is most popular?” and “Who is least popular?” For each student, the proportion of peer nominations received for ‘least popular’ was subtracted from the proportion of peer nominations received for ‘most popular’, to obtain a single continuum of popularity (Lease et al. 2002; Cillessen and Rose 2005). Popularity norms for aggression at T1 and T3 were calculated for each classroom as the correlation between peer-nominated aggressive behavior and popularity (Henry et al. 2000; Laninga-Wijnen et al. 2017). We transformed these variables into Fisher *z*-scores in order to obtain a relatively normally distributed measure, with the formula: *z’* = .5[*ln*(1 + *r*) – *ln*(1-*r*)] (Fisher, 1925).

#### Popularity Hierarchy

The popularity hierarchy at T1 was based on the standard deviation of individual popularity scores within the classroom. A high score reflects a strong classroom hierarchy, whereas a low score reflects a relatively egalitarian classroom.

#### Number of (Non-)Socially Dominant Aggressive, Prosocial, and Bi-Strategic Students

For each classroom, we calculated how many socially and non-socially dominant aggressive, bi-strategic and prosocial adolescents were present, and based on these numbers we calculated *percentages* of different types of students within each classroom (Table 2), in order to take classroom size into account.

**Table 2:** Description of the student types and norms *(N*_*classrooms*_ *= 120)*

	*M*	*SD*	Min %	Max %	Number of classrooms with this type of students
% Socially dominant highly aggressive	8.9	7.1	0	33.3	99
% Non-socially dominant highly aggressive	11.7	13.0	0	76.9	94
% Non-socially dominant bi-strategic	1.5	4.4	0	30.8	22
% Socially dominant bi-strategic	4.1	5.8	0	29.4	55
% Socially dominant highly prosocial	7.0	9.5	0	52.9	72
% Non-socially dominant highly prosocial	14.1	14.3	0	66.7	89
Aggressive popularity norm (correlation) T1	0.36	0.28	−0.52	0.81	
Aggressive popularity norm (Fisher *Z*-score) T1	0.41	0.34	−0.58	1.14	
Aggressive popularity norm (correlation) T3	0.39	0.24	−0.31	0.90	
Aggressive popularity norm (Fisher *Z*-score) T3	0.45	0.31	−0.32	1.45	
Popularity Hierarchy T1	.28	.07	.10	.44	

#### Analytic Strategy

As some students joined the school a year later, or left the school halfway through the year, there were some missing values in peer nominations (*N* = 29 at T1 and *N* = 27 at T3). Students with missing data at T1 were on lower educational tracks [*F*(1) = 5.42, *p* = .020] but did not differ with respect to age and sex. Students missing on T3 were a bit older [*F*(1) = 8.43, *p* = .004] but did not differ with respect to sex or educational track.

Moreover, our sample consisted of participants from three schools, which we had to combine in order to have sufficient power for our classroom-level analyses. Before doing so, we checked whether the schools were similar in terms of study variables. This was the case, except that one school was characterized by lower levels of peer-perceived aggression and a lower percentage of non-socially dominant aggressive adolescents compared to both other schools, and a lower percentage of non-socially dominant bi-strategic students compared to one of the other schools. The other two schools did not differ from each other. In addition, correlations between our main variables were transformed into *z*-scores and compared across different schools, and showed no differences. We therefore considered it justified to collapse the various groups for our analyses.

To examine the role of the percentage of socially and non-socially dominant aggressive, bi-strategic, and prosocial adolescents in the aggressive popularity norm, we conducted a longitudinal classroom-level linear regression analysis in M*plus* using maximum likelihood estimation (ML; Byrne, 1998), and using the BCBootstrap procedure to estimate indirect effects. Residuals were relatively normally distributed, there was no multicollinearity (Tolerance > .43 and VIF < 2.33), nor were there any serious outliers. We centered our student-type predictor variables and computed two interaction terms to examine potential moderating effects (percentage of socially dominant prosocial adolescents * percentage of socially dominant aggressive adolescents; and percentage of socially dominant prosocial adolescents * percentage of bi-strategic adolescents). Non-significant interaction effects were excluded from the final model. The percentage of different types of students, interaction terms and control variables at T1 were included as predictors of popularity norms at T1 and popularity norms at T3. We used indirect effects to test whether percentages of different student types *indirectly* predicted popularity norms at T3, via their effect on norms at T1. We also examined the potential mediating role of popularity hierarchy at T1 in predicting popularity norms at T3.

Model fit precision was examined using the chi-square statistic (χ^2^), comparative fit index (CFI), Tucker-Lewin Index (TLI), Root-Mean-Square Error of Approximation (RMSEA), and the Standardized Root-Mean-square Residual (SRMR). The χ^2^ test assesses the discrepancy of fit between the observed and hypothesized models; a non-significant χ^2^ value indicates a good fit to the data, but it should be noted that this test is overly sensitive to sample size and model complexity. The CFI and TLI estimates compare the specified model with a model in which all variables are assumed to be uncorrelated; values of .95 or greater reflect an excellent fit to the data, and values of .90–.94 indicate an adequate fit. The RMSEA index adjusts for model complexity and favors the most parsimonious model. RMSEA and SRMR values of .05 or less indicate an excellent fit to the data, and values of .06–.08 indicate an adequate model fit.

## Results

Individual-level correlations between aggression and prosocial behavior were significantly negative (*r =* −.10), whereas correlations of social dominance with aggression and prosocial behavior were significantly positive, with *r* = .45 and *r* = .27, respectively. Based on *z*-standardized scores of these three variables (aggression, prosocial behavior, and social dominance), we identified six types of students, which are represented in Fig. 1. We tested differences in unstandardized aggression, prosocial behavior and social dominance between the six student types; see Table 1. Table 2 provides information on how the six student types were distributed at classroom level. Most classrooms contained at least two of the different types of students, resulting in a large variety of possible combinations of students within classrooms. For the sake of parsimony, we did not report all possible combinations of student types within classrooms (available on request from the first author).

### The Role of Socially and Non-Socially Dominant Aggressive, Prosocial and bi-Strategic Adolescents in Popularity Norms and Popularity Hierarchy

**Fig. 2:**
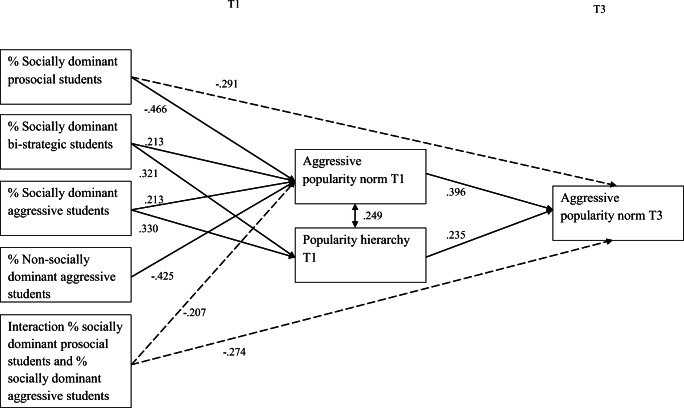
Significant standardized coefficients of class-level mediational regression analyses predicting aggressive popularity norms. Demographic variables and % of non-socially prosocial and bi-strategic adolescents were not visualized in this model. Significant direct effects are indicated with dotted lines, significant indirect effects are indicated with solid lines

In order to examine whether percentages of socially- and non-socially dominant aggressive, bi-strategic and prosocial adolescents were associated with aggressive popularity norms at T1 and T3, we conducted class-level linear regression analyses in M*plus*. As the interaction effect ‘percentage of socially dominant prosocial adolescents * percentage of bi-strategic adolescents’ was non-significant (in predicting norms at both T1 and T3) and contributed to a worse model fit [ΔRMSEA = .06; ΔCFI = .04; ΔTLI = .26; ΔSRMR = .01], we excluded this effect from our model. The fit of the resultant final model was good [χ^2^(5) = 5.47, *p* = .36; RMSEA = .03, CFI = .996; TLI = .973; SRMR = .016]. Figure 2 depicts significant standardized coefficients. Appendix 1 (Table 3) provides a complete overview of the results.

#### Popularity Norms at T1

As hypothesized, we found that higher percentages of socially dominant aggressive and socially dominant bi-strategic adolescents in a classroom were significantly associated with relatively higher aggressive popularity norms at T1, whereas a higher percentage of socially dominant prosocial adolescents was significantly associated with relatively lower aggressive popularity norms at T1. The percentage of non-socially dominant prosocial and bi-strategic students did not significantly predict aggressive popularity norms at T1. Contrary to our expectation, non-socially dominant aggressive students did contribute to the popularity norm, but in a reverse direction: a higher percentage of these students predicted *lower* aggressive popularity norms. The effect of the role of these non-socially dominant aggressive individuals was almost twice as great as the effect of socially dominant aggressive individuals.

**Fig. 3:**
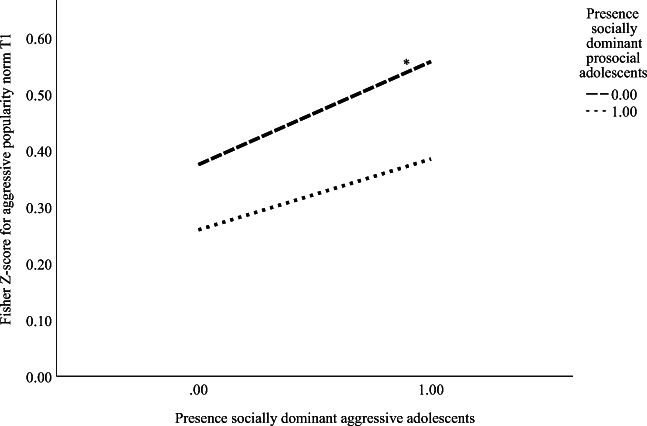
Interaction effect of the presence of socially dominant prosocial and aggressive adolescents in predicting aggressive popularity norms at T1 (*N*_classrooms_ = 120). Note. 0 = classrooms with <10.0% of socially dominant prosocial and aggressive students; 1 = classrooms with ≥10.0% of socially dominant prosocial and aggressive students. *p < .05

The percentage of socially dominant prosocial adolescents moderated the association between the percentage of socially dominant aggressive adolescents and the popularity norm at T1. Simple slope analysis showed that the aggressive popularity norm was highest in classrooms with a relatively high percentage of socially dominant aggressive adolescents and classrooms with a relatively low percentage (or even no) socially dominant prosocial adolescents present (Fig. 3). In total, 37.1% of the variance in aggressive popularity norms at T1 was explained by our model.

#### Popularity Hierarchy at T1

A higher percentage of socially dominant aggressive and socially dominant bi-strategic individuals was associated with a higher aggressive popularity hierarchy, whereas no relationship was found between the percentage of socially dominant prosocial individuals and the popularity hierarchy. None of the non-socially dominant student types contributed to the popularity hierarchy (Table 3, Appendix). In total, 28.1% of the variance in popularity hierarchy was explained by our model.

#### Popularity Norms at T3

**Fig. 4:**
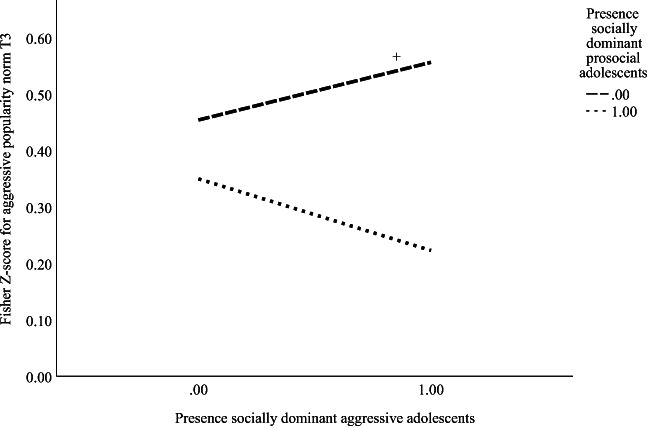
Interaction effect of the presence of socially dominant prosocial and aggressive adolescents in predicting popularity norms at T3 (*N*_classrooms_ = 120). Note. 0 = classrooms with <10.0% of socially dominant prosocial and aggressive students; 1 = classrooms with ≥10.0% of socially dominant prosocial and aggressive students. ^+^ *p* ≈ .05

After controlling for the popularity norm and popularity hierarchy at T1, most student types did not add to popularity norms at T3, except for a significantly negative main effect of the percentage of socially dominant prosocial individuals [*B* = −.010; *SE* = .004; *p* = .02], and a significant interaction effect (percentage of socially dominant prosocial adolescents at T1 * percentage of socially dominant aggressive adolescents at T1). Simple slope analysis revealed that classrooms with a relatively higher percentage of socially dominant aggressive adolescents were characterized mainly by higher aggressive popularity norms, if there were relatively fewer (or even no) socially dominant prosocial adolescents present (Fig. 4). Finally, we found that popularity norms were highly stable and that a stronger popularity hierarchy at T1 predicted higher aggressive popularity norms at T3. In total, 37.2% of the variance in aggressive popularity norms at T3 was explained by our model.

#### Indirect Effects

In the indirect part of our model, we tested whether the percentage of socially and non-socially dominant prosocial, aggressive and bi-strategic individuals would contribute *indirectly* to popularity norms at the end of the school year (T3), by setting the popularity norm at the start of the school year (T1). In addition, we examined potential mediating effects of popularity hierarchy.

We found six significant *indirect effects* (shown by *solid arrows in* Fig. *2*). The percentage of socially dominant aggressive individuals indirectly predicted higher aggressive popularity norms at T3 by contributing to higher popularity norms at T1 [*B* = .004, *CI*_bcbootstrap_ = .001–.008, *β* = .085]. We found a similar indirect effect for socially dominant bi-strategic individuals [*B* = .005, *CI*_bcbootstrap_ = .001–.012, *β* = .084]. Next, the percentage of socially dominant prosocial and non-socially dominant aggressive individuals indirectly predicted lower aggressive popularity norms at T3 by contributing to lower aggressive popularity norms at T1 [*B* = −.006, *CI*_bcbootstrap_ = −.012–-.002, *β* = −.185; *B* = −.004 *CI*_bcbootstrap_ = −.008 – -.001, *β* = −0.168]. This indicates that different student types contribute to the popularity norm at the start of the school year, and that their effect persisted due to the self-sustainability of norms.

Next, a higher percentage of socially dominant aggressive and bi-strategic individuals indirectly predicted higher aggressive popularity norms at T3 by contributing to a stronger popularity hierarchy at T1 [*B* = .003, *CI*_bcbootstrap_ = .001–.008, *β* = .077; B = .004, CI_bcbootstrap_ = .001–.011, *β* = .075]. Other effects reflecting the mediating role of popularity hierarchy were non-significant.

#### Classroom Demographic Characteristics

None of the classroom demographics were predictive of popularity norms or popularity hierarchy, except that higher school years were characterized by a stronger popularity hierarchy [*B* = .027, *β* = .241].

#### Sensitivity Analysis

We performed several sensitivity analyses. First, as the group of non-socially dominant bi-strategic adolescents was relatively small (*N*students = 36, distributed across 22 classrooms), we analyzed our models with *and* without the percentage of this student type, and all results remained the same. Accordingly, we chose the model including all student types as the final model. Second, we explored whether non-socially dominant aggressive students might moderate the role of socially dominant aggressive and bi-strategic students in the popularity norm (at both T1 and T3), but these interaction effects were non-significant and were therefore excluded from the final model. Third, we tested whether the results would be similar when controlling for the school or cohort, and this proved to be the case. The results of our sensitivity analyses are available on request.

## Discussion

This study examined whether the classroom student composition matters for the aggressive popularity norm. We found that a higher percentage of aggressive students predicted higher aggressive popularity norms, but only when these aggressive students were socially dominant. By contrast, non-socially dominant aggressive students contributed to *lower* aggressive popularity norms. Despite their highly prosocial behavior, socially dominant bi-strategic students enhanced the aggressive popularity norm; and only the socially dominant prosocial students who *abstained from aggression* lowered the aggressive popularity norm. Moreover, these socially dominant, solely prosocial students acted as a buffer against the role of socially dominant aggressive adolescents in the aggressive popularity norm (moderation), but not against the role of socially dominant bi-strategic adolescents. Finally, one way in which socially dominant aggressive and socially dominant bi-strategic individuals strengthen the aggressive popularity norm is by enhancing a classroom’s popularity hierarchy; these asymmetries in popularity may result in higher aggressive popularity norms, possibly due to enhanced competition for high status (Garandeau et al. 2014).

### The Number and Strength of Aggressive, Prosocial and bi-Strategic Individuals

In line with Moscovici’s theory on minorities (1974), our findings indicate that a numerical *minority* of students can determine the popularity norm in a classroom. However, somewhat counter to SIT (Latané and Wolf 1981), we found that it is not only *socially dominant* adolescents who may have the power to shape classroom-level aggressive popularity norms: Even when highly aggressive adolescents *lack* strength (social dominance), they contributed to the norm. That is, they *lowered* the aggressive popularity norm, with effect sizes that were almost twice as large as the effect of socially dominant aggressive individuals. Non-socially dominant aggressive students are at the periphery of the peer group and may be less attractive to their classmates, resulting in active rejection of their aggression (Chang 2004; Farmer et al. 2003; Sijtsema et al. 2010). They may also use their aggression in a less strategic and instrumental way compared to socially dominant aggressive individuals. Non-socially dominant aggressive students have been shown to experience more victimization (Hopmeyer Gorman et al. 2011; Farmer et al. 2003). In response, they may display aggression in a *reactive* rather than in a proactive way, which may be less attractive to others (Farmer et al. 2003; Prinstein and Cillessen 2003). Consequently, non-socially dominant students may provide a role model for how *not* to behave, and hence they may mitigate the value of aggression in the classroom context, resulting in lower aggressive popularity norms.

Next, we found support for *cross-*behavior processes: students’ endorsement of prosocial behavior predicted aggressive popularity norms; however, the role of this prosocial behavior depended on the kind of student using it. First, we identified a group of socially dominant bi-strategic individuals, who – compared to other student types – constituted the smallest minority in the classrooms. This numerical minority nevertheless seemed to be powerful: they contributed to the aggressive popularity norm over and above the role of socially dominant aggressive adolescents. Being prosocial in addition to being aggressive and socially dominant may thus provide *additional* power to enhance the aggressive popularity norm. The social skills of socially dominant bi-strategic adolescents may enable them to respond adeptly to social cues, ‘read’ their effect on peers, and create successful alliances (Hawley 2003). Rather than a voluntary act aimed at benefiting others (Eisenberg et al. 2009), therefore, the prosocial behaviors of these ‘well-adapted Machiavellians’ may be a self-serving strategy that secures their position and enhances their power to establish a norm for aggression (Hawley 2003).

In addition to these bi-strategic students, we identified a group of socially dominant prosocial adolescents who abstained from aggression, and hence were *solely* prosocial. The number of these solely prosocial students contributed to lower aggressive popularity norms. Classrooms with relatively more socially dominant prosocial individuals may represent a safe, harmonious environment (Jennings and Greenberg 2009) where behaviors serving the good of others may have valence and where aggression is perceived as inappropriate or non-adaptive (Chang 2004). This may decrease the salience and valence of aggression (Ellis et al. 2016). Moreover, the percentage of socially dominant prosocial adolescents in a classroom acted as a buffer against the role of socially dominant aggressive adolescents in the aggressive popularity norm (moderation). It might be that these socially dominant prosocial adolescents decrease the valence of aggression by showing that prosocial behavior can also be an effective means of gaining access to valuable material and social resources (Hawley 2003; Ellis et al. 2016). Socially dominant prosocial adolescents could not provide a buffer against the role of socially dominant bi-strategic adolescents in the aggressive popularity norm. One reason for this finding could be that bi-strategic individuals may indicate that being aggressive does not preclude being prosocial, and that the combination of behaviors may be the most effective for gaining access to resources (Ellis et al. 2016; Hawley, 2009).

As hypothesized, *no* relationship was found between the number of non-socially dominant prosocial and bi-strategic adolescents and the aggressive popularity norm. This is in line with Social Impact Theory (Latané and Wolf 1981), which posits that individuals may only have the power to contribute to the norm if their number and strength are relatively high. It should nevertheless be noted that power issues may have prevented us from detecting significant results for the relatively small group of non-socially dominant bi-strategic individuals (*N* = 36).

The number and strength of different types of students mattered particularly at the start of the school year. After controlling for popularity norms at T1, most student types did not add to variance in popularity norms at the end of the school year (T3) – except for the direct role of the percentage of socially dominant prosocial students and their moderating role in reducing the effect of socially dominant aggressive students on norms. Nevertheless, the role of socially dominant prosocial, aggressive, and bi-strategic, and non-socially dominant aggressive student types in popularity norms at T3 appears to be *indirect*: these students set the norm at the start of the school year, and their effect persists due to the stability of the norms (Laninga-Wijnen et al. 2018).

### Popularity Hierarchy as Underlying Mechanism for how Different Types of Students Contribute to Aggressive Popularity Norms

We found that one way in which socially dominant aggressive and socially dominant bi-strategic individuals contribute to higher aggressive popularity norms is by enhancing the classrooms’ *popularity hierarchy*, probably because they use their aggression in a strategic, manipulative way, which allows them to gain a higher status in the peer group *at the expense of* others’ status. In line with the balance of power perspective (Garandeau et al. 2014), we found that these strong popularity asymmetries in turn predict higher aggressive popularity norms. Popularity asymmetries may evoke a power imbalance, which facilitates abuse of power through aggression among popular peers (Garandeau et al. 2011). Moreover, when all the benefits associated with being popular are not equally available to everyone (Hawley 2003), this may trigger competition for popularity. In a competitive context, aggression may be seen as a valuable tool for gaining or maintaining popularity (Laninga-Wijnen et al. 2019). Importantly, we found no relationship between other types of students and popularity hierarchy, which partly contradicts our hypotheses as we initially expected a higher number of socially dominant prosocial individuals to contribute to a lower popularity hierarchy. Apparently, prosocial behaviors, even when displayed by socially dominant adolescents, may have no effect on classroom popularity asymmetries, most likely because these behaviors are aimed at benefitting others and therefore more important to dyadic, liking relationships, rather than to reputation-based constructs such as popularity or popularity hierarchy (Hopmeyer et al. 2011).

### Classroom Demographic Characteristics

We found no evidence that classroom demographics predict aggressive popularity norms, which was somewhat counter to our hypotheses; the aggressive popularity norm does not depend directly on classroom size, sex proportion, educational level, or school year. This suggests that in order to prevent the emergence of aggressive popularity norms, schools may need to look beyond demographics such as classroom size or sex proportion, and focus instead on the type of individuals making up a class. This may be most important in the most senior school years, which are more likely to be characterized by a strong popularity hierarchy, which in turn is associated with higher aggressive popularity norms.

### Strengths, Limitations and Future Directions

Our study has several strengths. First, whereas previous studies have focused on the *consequences* of aggressive popularity norms (Laninga-Wijnen et al. 2018), we examined which factors may *predict* aggressive popularity norms in the first place. Second, our study sheds new light on Social Impact Theory and Resource Control Theory. We demonstrated that aggressive students who *lack* strength may still matter for the aggressive popularity norm, and that *cross-*behavior processes may occur: the endorsement of certain behaviors (prosocial behavior) may affect the norms regarding *other,* related behaviors (aggression). Importantly, our generalized measures of prosocial and aggressive behaviors only allowed us to test aspects of resource control theory indirectly, as we did not directly assess the *function* of these behaviors (e.g. the instrumentality; Hawley and Bower 2018; p. 106). Third, we identified the popularity hierarchy as a mechanism explaining *why* socially dominant aggressive and bi-strategic individuals may have the power to set the norm. Accordingly, this study not only provides information on the types of students that matter for the aggressive popularity norms, but also on *why* they may have the power to set the norm.

Some limitations of the present study need to be acknowledged. First, the data used in the current study stem from peer nominations only, which might lead to problems with shared method variance (Vaillancourt and Hymel 2006); to counter this, measures were aggregated across multiple nominators, enhancing the validity and reliability (Bukowski et al. 1993; Bukowski and Hoza 1989). Second, we examined peer-reported aggression as a unified construct, without consideration for its different forms (i.e. physical vs. relational) and functions (i.e. reactive vs. proactive). Nonetheless, the four items we used loaded reliably on one factor. We would encourage researchers to disentangle different types of aggression. Third, we cannot make any statements about causality. It might also be the case that, due to the presence of aggressive popularity norms at T1, highly prosocial students are less often seen as a leader at T1, whereas highly aggressive adolescents may attain social dominance. Fourth, we collapsed three different schools in order to obtain sufficient power for our classroom-level analyses. One school differed somewhat from the other two schools, but additional analyses indicated that these differences did not affect our results. Nevertheless, we would advocate further research involving more schools and classes to replicate our analyses in order to determine whether similar results are obtained, and to examine potential moderating effects of classroom demographic characteristics. Fifth, in line with some other studies (Hawley 1999; McDonald et al. 2015) we relied heavily on theory to identify our six student types, rather than using a data-driven approach such as Latent Profile Analyses. We would encourage future research to examine the use of these types of adolescents further by comparing it to outcomes of more data-driven approaches.

### Conclusions and Implications

Our findings have important implications for theory and practice, as they provide a first step in examining predictors of aggressive popularity norms by focusing on classroom composition. First, schools could use the insights generated by this study to *prevent* the emergence of aggressive norms, perhaps starting with decision-making on classroom composition. In other words, schools could base classroom composition more actively on the combination of student types within classrooms. Schools might obtain information on students’ behavior or social dominance from primary schools or from observations in previous years at secondary school, and this information could help them organize classrooms in such a way that the percentage of socially dominant aggressive adolescents and socially dominant bi-strategic students is kept to a minimum, while the percentage of socially dominant prosocial students is maximized across classrooms.

Second, the knowledge gained in this study can be used to propose solid research-based intervention strategies designed to *change* aggressive popularity norms. Some interventions, such as the Meaningful Roles Intervention (Ellis et al. 2016) or Roots Intervention (Paluck et al. 2016), are aimed at reducing aggressive (bullying) norms by rewarding prosocial behavior, for example by assigning prosocial leaders in a classroom and exchanging compliment cards. Our study may provide preliminary theoretical evidence for the potential effectiveness of such interventions, as well as suggestions for improvement. For instance, in line with the reasoning of the Meaningful Roles Intervention, we confirmed that socially dominant prosocial students have the power to diminish the detrimental role of socially dominant aggressive individuals. However, we also found indications that it may be important to provide additional strategies to actively discourage aggressive behavior: our study shows that adolescents who combine prosocial behavior with aggression still contribute to higher aggressive popularity norms. Also, when aggression goes together with being *non-*socially dominant, this may diminish the value of aggression in a given context, resulting in a lower aggressive popularity norm. Future research should examine what means may be most effective in discouraging aggression in appropriate ways. In addition, future researchers are encouraged to examine how socially dominant bi-strategic students may be constrained in setting an aggressive popularity norm, as their effect was not buffered by socially dominant prosocial adolescents. Future studies might also examine the origins of prosocial popularity norms, as it has been suggested that these norms yield more desirable classroom environments. This would enable more insights to be gained into how the classroom composition may contribute to environments that appropriately foster early adolescents’ socio-emotional and academic adjustment.

## Appendix

**Table 3:** Results of longitudinal class-level regression analysis predicting aggressive popularity norms at T1 and T3 and popularity hierarchy at T1 *(N*_*classrooms*_ *= 120)*

	Aggressive popularity norms T1			Popularity hierarchy T1			Aggressive popularity norms T3		
	*B*	*SE*	*β*	*B*	*SE*	*β*	*B*	*SE*	*β*
Percentage socially dominant aggressive students	0.010*	0.004	0.213	0.003***	0.001	0.330	−0.006	0.004	−0.139
Percentage socially dominant bi-strategic students	0.012*	0.006	0.213	0.004**	0.002	0.321	−0.001	0.005	−0.019
Percentage socially dominant prosocial students	−0.017***	0.004	−0.466	0.000	0.001	0.045	−0.010**	0.004	−0.291
Percentage non-socially dominant aggressive students	−0.011***	0.003	−0.425	0.000	0.001	0.012	−0.002	0.002	−0.084
Percentage non-socially dominant bi-strategic students	0.004	0.007	0.049	0.001	0.001	0.072	0.006	0.008	0.086
Percentage non-socially dominant prosocial students	−0.003	0.003	−0.134	0.001	0.001	0.098	−0.002	0.002	−0.099
Interaction socially dominant aggressive * socially dominant prosocial students	−0.001*	0.000	−0.207	–	–	–	−0.001**	0.000	−0.274
Popularity hierarchy T1	0.004*	0.002	0.249	–	–	–	1.014**	0.405	0.235
Aggressive popularity norm T1	–	–	–	0.004*	0.002	0.249	0.369**	0.124	0.396
Class size	0.010	0.008	0.145	−0.001	0.002	−0.082	–	–	–
Sex proportion	0.142	0.215	0.052	0.006	0.058	0.011	–	–	–
Educational level	0.103	0.072	0.150	0.001	0.014	0.007	–	–	–
Grade	−0.009	0.043	−0.018	0.027*	0.011	0.241	–	–	–
